# Tribological Characterization of an Epoxy Composite Coating for Enhanced Wear Resistance in Oil Well Casing Applications

**DOI:** 10.3390/polym17162192

**Published:** 2025-08-11

**Authors:** Ahmad Bawagnih, Necar Merah, Fadi Al-Badour, Mohammed Abdul Azeem, Amjad Shaarawi, Abdulwahab Aljohar, Ali Hijles

**Affiliations:** 1 Department of Mechanical Engineering, King Fahd University of Petroleum and Minerals, Dhahran 31261, Saudi Arabia; g202308850@kfupm.edu.sa (A.B.); fbadour@kfupm.edu.sa (F.A.-B.); maazeem@kfupm.edu.sa (M.A.A.); 2 Interdisciplinary Research Center for Advanced Materials, King Fahd University of Petroleum and Minerals, Dhahran 31261, Saudi Arabia; 3 Drilling Technology Team, EXPEC Advanced Research Center, Saudi Aramco, Dhahran 31261, Saudi Arabia; amjad.shaarawi@aramco.com (A.S.); abdulwahab.aljohar@aramco.com (A.A.); ali.hijles@aramco.com (A.H.)

**Keywords:** epoxy composite coating, nanofillers, specific casing wear rate, tribofilm

## Abstract

The tribological performance of a novel nonmetallic composite casing coating is investigated under dry wear conditions and different side loads and rotational speeds. The coating is composed of a short-glass-fiber-reinforced epoxy matrix with silicon carbide, aluminum oxide, and calcium carbonate nanofillers to provide a protective barrier against contact with hardened drill pipe tool joints. The results revealed that the wear behavior was highly dependent on the applied side load and rotational speed. Under high-load conditions, the formation of a compacted tribofilm significantly reduced the coefficient of friction and specific wear factor by limiting direct surface contact. Lower rotational speeds and moderate side loads resulted in adhesive wear with formation of stable tribofilms that mitigated material loss.

## 1. Introduction

In oil and gas well drilling, wear between the casing material and the hardened drill pipe tool joint (DP-TJ) is an inevitable issue that can, in severe regimes, result in deformed or even collapsed casings and fractured drill pipes. The drill pipe tool joint is 20% to 30% larger than the drill pipe body, which increases its exposure to mechanical contact with the inner surface of the casing. Failing to address this issue can significantly drive up costs and may force the abandonment of the well before reaching its target depth [1,2]. Casing wear is influenced by several key factors, including drilling depth, duration of drilling operations, the contact load between the casing and drill pipe, the rotation speed of the DP-TJ, and the material of the casing itself. Each of these factors significantly influences the level of wear encountered, affecting the overall integrity and lifespan of the well structure [3]. Various carbon steels are used in drilling and casing applications, valued for their good mechanical properties and durability. The most prominent grades include J55, K55, L80, N80, C95, and P110. These grades are selected based on their unique mechanical and tribological properties, tailoring them to the specific demands and wear resistance required by each well [4,5]. Various approaches exist for analyzing metal casing wear beyond standard testing methods. These range from finite element analysis (FEA) to predict the wear depth and coefficient of friction (COF) to physical experiments that simulate real-world casing wear conditions in oil well drilling [2,6,7].

In other studies, custom-built experimental setups utilizing modified lathe machines were developed to simulate real casing wear during the wellbore drilling process [8]. These setups employed actual casing materials and incorporated stepper motors to precisely control the applied side load, effectively replicating the mechanical interaction between the drill pipe and the casing. The wear depth was monitored using a digital micrometer and validated through 3D optical profilometry.

A casing wear factor is employed by many researchers as a means to predict casing wear because it provides a convenient and relatively straightforward method to estimate the amount of wear based on operational parameters. While the precise mechanisms of casing wear are complex and involve multiple interacting factors such as material properties, drilling fluid characteristics, contact forces, etc., a wear factor offers a simplified approach to quantify the cumulative wear effect by correlating it to drilling parameters such as contact pressure, sliding distance, and rotational speed [9]. Polymers and polymer composites have recently gained acceptance as substitutes for metals due to their excellent corrosion resistance. They are lighter, which reduces transportation and handling costs and facilitates installation in deep or extensive wells where weight is crucial [10,11,12]. Despite their advantages, these materials face challenges such as higher thermal expansion, shorter durability, and sensitivity to high temperatures, demanding thoughtful design efforts [13]. The development of casing materials now focuses on achieving a balance between light weight, cost-effectiveness, and effective wear mitigation throughout the drilling process. Polymer-based composite casing linings present a viable alternative when considering tribological properties. These composites offer benefits such as self-lubrication, lower density compared to metal materials, and a low coefficient of friction [14,15]. However, a low COF does not necessarily correlate with a low wear rate, as the wear rate can be influenced by various operational conditions, including temperature, load, velocity, and others [16]. Li et al. [17] investigated the tribological behaviors of pure polyetheretherketone (PEEK) and PEEK composites reinforced with 30 wt.% short glass fibers (SGFs) using a ball-on-disc setup at room temperature. The results indicated that as the applied load and sliding time increased, both the friction coefficient and wear of the SGF/PEEK composite gradually increased and eventually stabilized. Notably, the SGF/PEEK composite demonstrated enhanced wear resistance compared to pure PEEK. The authors attributed this enhanced tribological property to the greater thermal stability of the SGF-reinforced composite, resulting in a lower weight loss at high temperature compared with pure PEEK. Birleanu et al. [18] investigated the tribological characteristics of glass-fiber-reinforced polymer (GFRP) composites using dry sliding friction tests performed via a ball-on-disc tribometer. Experimental analyses encompassed critical operational parameters, including the side load, rotational speed, and glass fiber weight percentage. The findings indicated a clear correlation between glass fiber content and tribological performance, wherein an increase in fiber concentration initially led to a reduced coefficient of friction (COF). However, beyond an optimal threshold, higher fiber fractions elevated the composite’s wear rate due to increased brittleness and fiber–matrix interface degradation. Specifically, a significant specific wear rate (K) of approximately 32.73 × 10^−5^ (mm^3^/N-m) was recorded at a 20 N load and sliding speed of 0.25 m/s with a maximum glass fiber content of 65.3 wt.%. Conversely, optimal tribological performance was identified under intermediate conditions (54 wt.% glass fiber), with a lower applied load of 10 N and sliding velocity of 0.1 m/s, thereby balancing frictional properties and wear resistance. These outcomes highlight the critical need to precisely optimize composite fabrication parameters, including the filler diameter, volume fraction, weight percentage, and matrix composition, to achieve enhanced multi-functional performance and durability of frictional composites in practical applications.

Various metallic nanofillers, such as copper (Cu) and iron (Fe), as well as nonmetallic oxides like copper oxide (CuO), zinc oxide (ZnO), titanium dioxide (TiO_2_), zirconium dioxide (ZrO_2_), silicon dioxide (SiO_2_), and silicon nitride (Si_3_N_4_), have been shown to significantly enhance the mechanical properties of polymer nanocomposites [13]. These fillers not only reduce the friction coefficient but also improve wear resistance and thermal properties [19]. This effectiveness is largely due to the larger surface area of nanoparticles compared to micro- or macroparticles, which aids in lowering the wear rate of the composite. In particular, nanoparticles within polymer composites demonstrate a distinct capability to form robust, resilient tribofilms when combined with wear debris under tribological conditions [20,21]. The integration of nanoparticles facilitates the formation of a compacted, stable surface layer, which significantly reinforces the wear interface, enhancing the durability and wear resistance of polymer nanocomposites. This tribofilm effectively acts as a protective barrier, reducing direct asperity contact, mitigating abrasive interactions, and contributing to prolonged component lifespan in demanding operating environments [22]. The wear behavior of polymers differs from that of metallic materials. In these composites, material removal during contact with a counter surface occurs through various mechanisms. The predominant mechanism is adhesive wear, characterized by the detachment of fine polymer particles from the surface, alongside fiber–matrix detachment and fiber failure. Conversely, the presence of metal asperities from the counterface or scratches at the counterface suggests that wear occurs more through abrasion than adhesion [23,24].

Basavarajappan et al. [25] examined the tribological performance of glass–epoxy (G–E) composites incorporated with silicon carbide (SiC) and graphite fillers under dry sliding conditions utilizing a pin-on-disc tribometer. Their study demonstrated that introducing fillers significantly enhanced the wear resistance of the G–E composites compared to unfilled variants. Nevertheless, a further increment in SiC content up to 10 wt.% adversely affected the wear performance, attributed primarily to increased abrasive interactions, leading to accelerated material degradation. Additionally, the tribological behavior was notably influenced by the formation and characteristics of the transfer film developed on the counterface surface during testing, which moderated frictional interactions and wear rates. This underscores the critical balance required in filler content optimization to achieve superior wear resistance without compromising the mechanical integrity of the composite. In another study, Bobbili and Madhu [26] examined the characteristics of E-glass–epoxy-reinforced composites and multiwalled carbon nanotubes (MWCNTs) in comparison to epoxy/MWCNT composites by sliding wear. They observed a reduction in both the COF and specific wear as the percentage of MWCNTs increased (ranging from 0 to 1.5 wt.%). Microscopic examinations of the worn sample fracture surfaces revealed that fiber debonding and fiber pull-out occurred when the stresses at the fiber–matrix interface exceeded the interfacial strength, leading to fiber detachment from the matrix. Additionally, Albahkali et al. [27] conducted a tribological study of epoxy-based hybrid composites with aluminum oxide (Al_2_O_3_) nanoparticles and graphite reinforcement at varying weight fractions, from 0 to 0.5 wt.%. Their friction tests showed a notable reduction in the COF and mechanical wear due to the enhancement in mechanical properties such as hardness and toughness with the addition of reinforcement material. Furthermore, the inclusion of graphite nanoplatelets in CFRP has shown a reduction in the COF and wear rate due to the formation of a tribofilm at the sliding surface. This film reduced the interfacial friction and lowered the shear resistance between contact surfaces, ultimately resulting in decreased wear in the nanocomposites [28]. Recently, Fouad et al. [7] characterized the wear of a zirconia-toughened epoxy/Kevlar honeycomb composite coating for steel casings under dry wear conditions at different side loads and DP-TJ rotational speeds. They found that the specific wear rate (K) varied with the speed increase at all loads, reaching a maximum of 20.3 × 10^−6^ mm^3^/Nm at 1200 N and then dropping to 8 × 10^−6^ mm^3^/Nm at 1400 N due to the formation of a double transfer layer of wear debris that prevented direct contact between the composite surface and DP-TJ.

This study investigates the tribological behavior and wear characteristics of a novel short glass fiber epoxy composite with nanofiller, intended as a coating for nonmetallic alternatives to traditional steel casings in drilling operations. Unlike previous research works on similar composites that relied on simplified pin-on-disc methods, this work employs real-size coated GFRE casings and actual drill pipes under realistic operating conditions. It analyzes the effects of the side load and drill pipe tool joint (DP-TJ) rotational speed on the wear depth, wear volume, specific wear rate, coefficient of friction (COF), and contact temperature. The thermal history during testing is recorded and used to explain the wear behavior of the present system. Surface characterization is also conducted to identify wear mechanisms and material compositions. To the authors’ knowledge, this is the first study to explore this specific composite coating in this specific setup. The findings aim to support the oil industry in considering lighter, cost-effective, and corrosion-resistant nonmetallic casing alternatives.

## 2. Materials and Methods

### 2.1. Material and Sample Preparation

**Figure 1:**
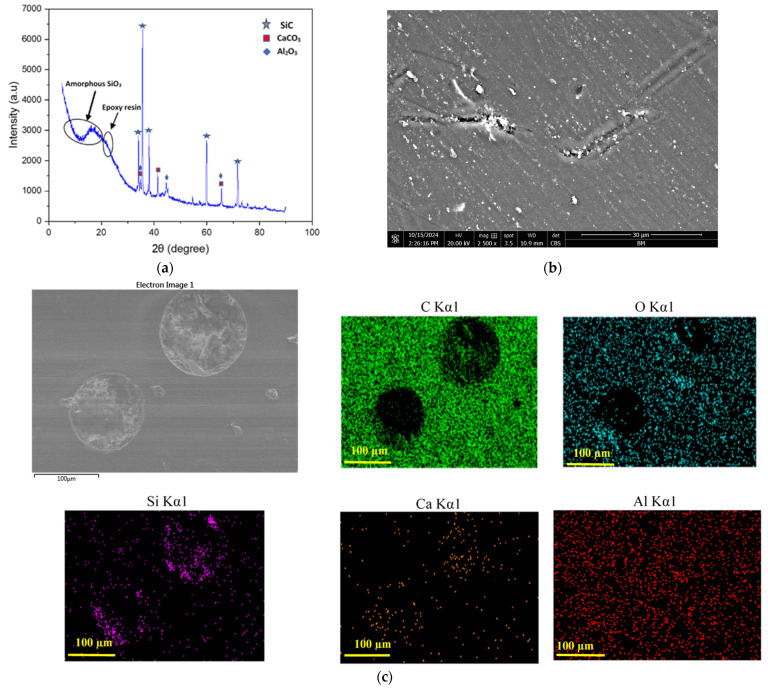
Characterization of the as-received epoxy composite coating: (**a**) XRD analysis of coating material component, (**b**) back-scattered SEM image of the coating surface, and (**c**) EDS elemental maps for coating material.

**Figure 2:**
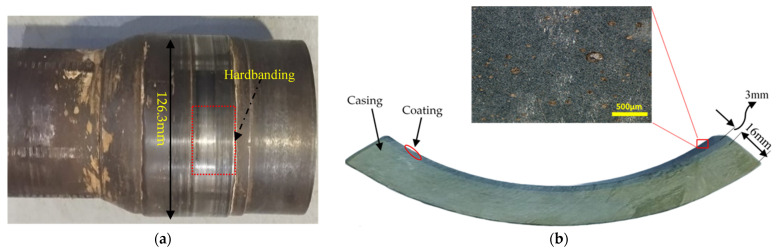
(**a**) Drill pipe tool joint (DP-TJ) used in well drilling operations and (**b**) casing lining sample.

The casing material used in this study comprised a continuous glass-fiber-reinforced epoxy (GFRE) composite, fabricated via a filament winding process. Continuous E-glass fibers were helically wound onto precision-machined steel mandrels to form the base structural casing. To improve tribological performance, particularly at the critical inner surface where contact with the DP-TJ occurs, a wear-resistant nonmetallic composite coating was applied with a layer thickness of approximately 2.5–3.0 mm to the internal surface of the 16 mm thick GFRE composite casing. This coating is composed of an epoxy resin matrix reinforced with short glass fibers (SGFs) and filled with ceramic nanoparticles. The primary fillers included silicon carbide (SiC), aluminum oxide (Al_2_O_3_), and calcium carbonate (CaCO_3_). The presence of these phases was confirmed by X-ray diffraction (XRD) analysis, as shown in Figure 1a, which displays characteristic peaks corresponding to crystalline SiC, Al_2_O_3_, and CaCO_3_, along with a broad hump related to the amorphous epoxy matrix and silica-based components. The surface morphology and filler distribution of the as-received composite before the test were further examined using SEM and EDS mapping, as shown in Figure 1b,c. The SEM image shows a relatively smooth layer of epoxy but also features voids, particle agglomeration likely caused by entrapped air, solvent evaporation, and localized shrinkage of the epoxy resin during curing. These defects could act as weak points during wear, affecting local film integrity. Short glass fibers were not visible at the surface; they were embedded beneath the outer epoxy layer. Elemental mapping confirms the uniform presence of Si, Al, Ca, C, and O, corresponding to SiC, Al_2_O_3_, CaCO_3_, and the organic resin. The distribution maps show good distribution of particles in the coating material. Fe was not detected, confirming that the coating surface was free of contamination prior to testing. The coating typically experiences intense mechanical interaction with DP-TJ hardbanding (HRC~50), as illustrated in Figure 2a. All coated casing samples were supplied by a local manufacturer, while the DP-TJ components were supplied by a local oil company. The coated composite GFRE pipes were sectioned into 60° arc-shaped samples to facilitate wear testing, as detailed in Figure 2b. The dimensions of both the composite casing and the tool joint are summarized in Table 1.

### 2.2. Friction and Wear Tests

**Figure 3:**
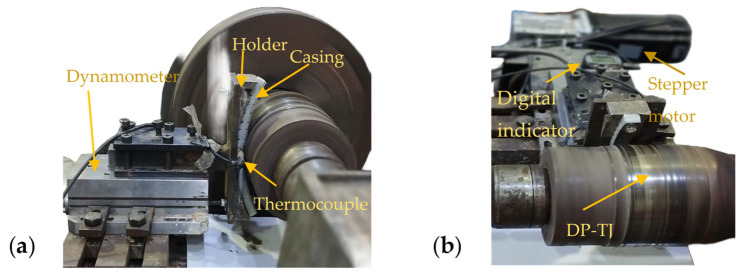
Wear test setup and components: (**a**) side view and (**b**) front view.

A custom-designed experimental apparatus (Figure 3), adapted from a modified lathe machine [9], was used to conduct controlled wear and friction tests on the prepared casing samples. The setup was configured in accordance with the API Standard 7CW (2015) [29], ensuring relevance to industry-standard testing conditions. This apparatus allowed for precise measurement of the wear depth, DP-TJ rotational speed, side load, and frictional forces while maintaining consistent operational parameters. The arc-shaped composite casing samples were rigidly mounted onto a custom-fabricated fixture. This fixture was firmly mounted on a three-axis dynamometer (Figure 3a), enabling real-time force acquisition in the X, Y, and Z directions. A stepper motor controlled by an HBS860 driver and Arduino microcontroller was employed to apply the required side load onto the rotating hardened steel DP-TJ. A closed-loop feedback system was integrated using the force data from the dynamometer to dynamically adjust the motor output, ensuring that the applied load remained stable within the target range throughout the test duration. This feedback mechanism ensured repeatability and accuracy of the wear conditions. Data acquisition was centralized through a microcontroller-based interface, which coordinated sensor readings and motor control in real time. Several parameters were continuously recorded, including the following:

- The bulk temperature of the casing sample was measured using a DS18B20 digital sensor embedded in a pre-drilled hole near the coating (Figure 3a), enabling continuous monitoring of thermal effects due to friction.
- The surface temperature at the casing–DP-TJ interface was monitored via a FLIR E8-XT infrared thermal camera (FLIR Systems, Wilsonville, OR, USA), which was positioned to capture thermal radiation directly from the wear zone.
- The wear depth was measured using a high-resolution electronic micron indicator (Figure 3b) with a least count of 1 μm and an accuracy of ±4 μm. Measurements were recorded at two-minute intervals to track the progression of material loss.
- The force components were captured by the dynamometer in real time, to characterize normal and tangential components influencing the wear mechanism.

The combination of multi-sensor integration, closed-loop load control, and high-frequency data logging provided a comprehensive dataset. This enabled an in-depth evaluation of the tribological behavior of the composite coating under realistic operational conditions, with particular attention to its performance against hardened steel tool joints under dry friction.

### 2.3. Experimental Procedure

The experimental design employed a factorial approach, systematically varying both the side load and the rotational speed of the DP-TJ to comprehensively investigate their influence on the wear behavior of the composite coating materials. Three distinct levels of side load (500 N, 700 N, and 1000 N) were selected to represent a range of contact pressures that might be higher than those encountered in actual drilling operations, which are typically around 0.3 to 0.5 MPa. Similarly, three DP-TJ rotational speeds (65 RPM, 115 RPM, and 154 RPM) were chosen to cover a range of typical drilling speeds.

To ensure the reliability and reproducibility of the results, each combination of side load and rotational speed (resulting in a total of nine distinct test conditions) was repeated two or three times. Each test was run for five hours, providing ample time for the wear mechanisms to fully manifest and to obtain statistically meaningful data. The selected side load and rotational speed levels are summarized in Table 2. Throughout the duration of each test, several key parameters were continuously recorded and used to estimate the specific wear rate, wear volume, and coefficient of friction.

### 2.4. Characterization

The hardness of the casing coating was measured, before and after the wear test, using Shore D hardness testing equipment (BAREISS, Baiersbronn, Germany), following the ASTM D2240 standard [30]. This provided a quantitative measure of the materials’ resistance to indentation, enabling the assessment of any changes in hardness due to wear. To characterize the microstructural changes and wear mechanisms, high-resolution digital microscopic images were acquired using a DSX-1000 digital microscope (Olympus IMS, Waltham, MA, USA) at various locations and angles on both as-received and worn casing samples. This allowed for detailed visualization of the nonmetallic composite coating material lining’s microstructure, providing valuable context for interpreting the wear data. Further investigation into the wear mechanisms was conducted using Quanta 250 scanning electron microscope (FEI Company, Hillsboro, OR, USA) coupled with Oxford energy dispersive X-ray spectroscopy (EDS) system (Oxford Instruments, Abingdon, Oxfordshire, UK). The EDS analyses determined the elemental composition at various points on the worn surfaces. This multifaceted approach, using different techniques, provided a comprehensive characterization of the wear mechanisms, complementing the wear test results and enabling a complete understanding of the wear behavior of this novel composite casing coating material.

## 3. Results and Discussion

### 3.1. Hardness

**Figure 4:**
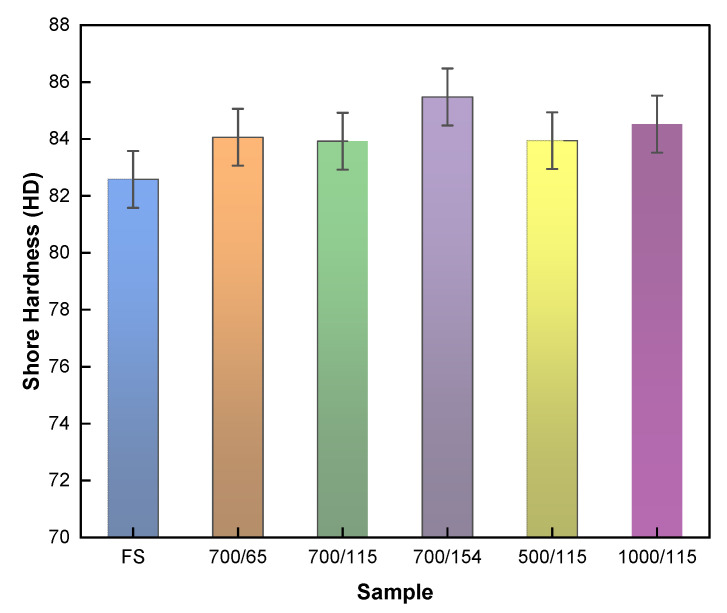
Average Shore D hardness results for the fresh sample (FS) and worn samples tested at different sliding conditions: 700 N load at 65, 115, and 154 rpm and 500 N and 1000 N loads at 115 rpm.

Initially, the hardness of the coating under investigation was evaluated before and after the test to comprehend the wear behavior. Figure 4 illustrates the average Shore D hardness of the casing coating samples before and after wear tests under various DP-TJ rotational speeds and side loads. Notably, most worn samples exhibited a modest increase in surface hardness, ranging between 2% and 4%, compared to the as-received coating sample (FS). The observed increase in hardness is likely due to surface densification mechanisms induced by tribological loading during the wear process. Under repeated mechanical loading and sliding friction, the worn surface experiences dynamic compaction of wear debris, which includes fragmented short glass fibers (SGFs), debris of epoxy matrix, and dispersed nanoparticles. These debris particles, subjected to high local pressures and frictional heat, are progressively embedded and compacted into the surface layer [31]. This compaction mechanism likely reduces surface porosity and leads to the formation of a dense, load-bearing tribolayer, thereby improving the resistance to indentation and increasing the Shore D hardness.

### 3.2. Wear Depth and Wear Volume

**Figure 5:**
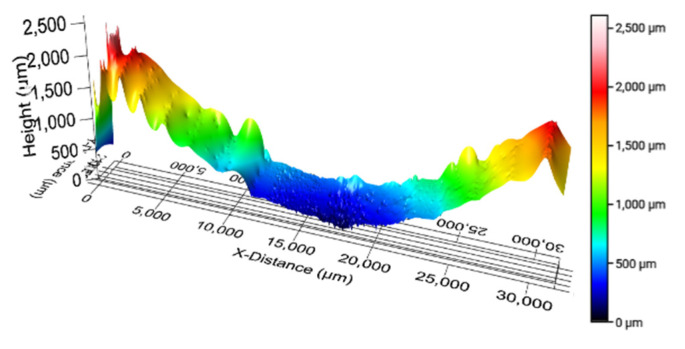
Three-dimensional tracking profile of worn casing at 500 N load and 154 rpm.

**Figure 6:**
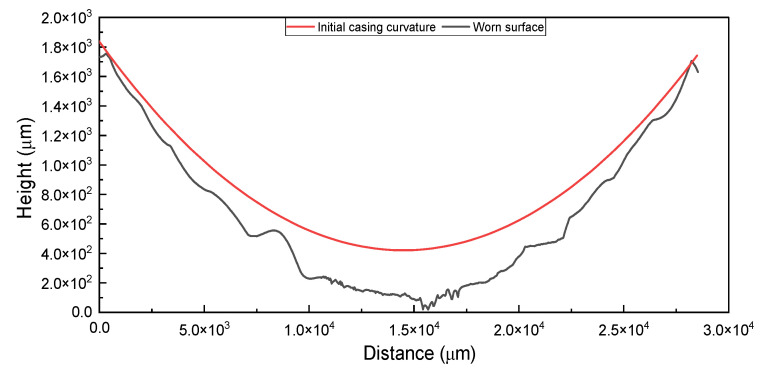
Two-dimensional wear tracking profile of worn casing at 500 N load and 154 rpm compared to unworn profile depicting crescent-shaped groove.

During the wear test, the wear depth was recorded at two-minute intervals using a high-precision digital displacement indicator. To validate the accuracy of these measurements, a 3D optical profilometer was employed to map the worn surface topography. As shown in Figure 5, the profilometric data enabled detailed tracking of the wear scar geometry, which was then compared against the initial casing curvature profile representing pre-test measurements in Figure 6. The comparison between the two methods showed that the in situ measurement was within 10% of that obtained from the profilometer. Hence, to estimate the wear volume, the maximum wear depth obtained from the digital indicator was used as a key parameter. Given that the observed wear cross-section resembles a crescent-like shape, a geometrical approach was adopted to calculate the volume of removed material, as reported in prior studies [32,33,34].

**Figure 7:**
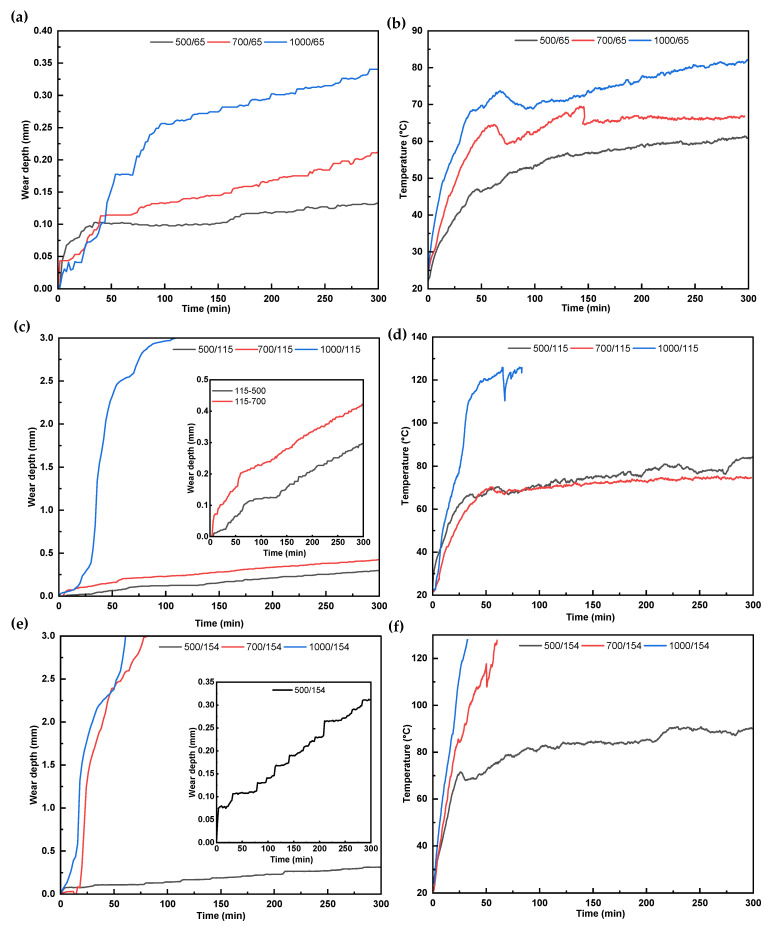
The wear depth versus time profile (left) and temperature versus time profile (right) (**a**,**b**) at 65 rpm and 500, 700, and 1000 N; (**c**,**d**) at 115 rpm and 500, 700, and 1000 N; and (**e**,**f**) at 154 rpm and 500, 700, and 1000 N.

Figure 7 illustrates the progression of wear depth and bulk temperature over a 5 h test duration under side loads of 500, 700, and 1000 N and DP-TJ rotational speeds of 65, 115, and 154 rpm. From the data obtained, it can be observed that, across all conditions, the wear depth increases rapidly during the first few minutes of the test. This is due to the typical initial layer of the coatings, mainly constituted of epoxy resin before exposing the harder nanofillers in the contact zone. This outer layer is more vulnerable to early-stage wear and material removal. Following this phase, both the wear depth and temperature curves begin to gradually increase. They tend to stabilize into a near-linear trend—particularly at 65 rpm across all side load levels, as shown in Figure 7a,b. A similar linear behavior is also evident at a moderate speed of 115 rpm under 500 N and 700 N loads, as shown in Figure 7c,d. Additionally, near-stable characteristics are observed at 154 rpm under a 500 N side load, as illustrated in Figure 7e,f.

**Figure 8:**
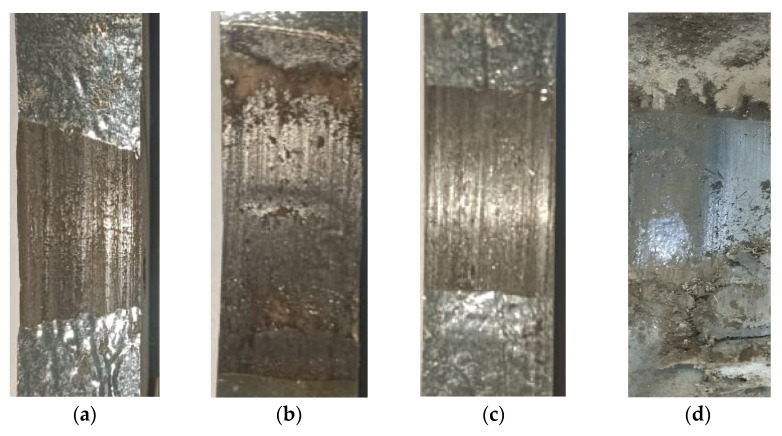
Digital image of worn surfaces at the end of the test at (**a**) 1000 N-65 rpm, (**b**) 1000 N-115 rpm, (**c**) 500 N-154 rpm, and (**d**) 1000 N-154 rpm.

In contrast, under more aggressive testing conditions, such as 115 rpm at 1000 N and 154 rpm at both 700 N and 1000 N (Figure 7c,e), a rapid and continuous increase in wear depth is recorded until the entire thickness of the composite coating is worn out. Concurrently, the temperature rises steadily in Figure 7d,f, ultimately exceeding 120 °C, which is near or beyond the glass transition temperature (T_g_) of the epoxy matrix [35]. Once the T_g_ is exceeded, the epoxy matrix softens significantly, losing its load-bearing capacity. This condition accelerates matrix degradation, fiber–matrix debonding, and fiber pull-out, resulting in severe wear behavior and, in extreme cases [36], thermal damage or burning of the substrate casing, as observed in high-rotational-speed, high-side-load tests specifically at 154 rpm under 700 and 1000 N. The influence of the rotational speed is further highlighted by comparing tests at a constant side load (e.g., 500 N) but with an increasing rotational speed (from 65 rpm to 154 rpm). A clear increase in the wear depth is observed with rotational speed, which is explained by the higher frictional heat generated at increased velocities. This heat contributes to surface softening of the matrix, reduces its ability to support embedded fibers and nanoparticles, and weakens the interfacial bonding, making the material more prone to rapid removal [37]. These thermal effects are evident from the temperature–time profiles, which show a direct correlation between rotational speed and temperature rise in Figure 7b,d,f. Additionally, increasing the side load from 500 N to 1000 N at the same rotational speed (e.g., 65 rpm or 115 rpm) results in a notable increase in the wear depth. Figure 8 presents photographic images of the worn surfaces of the tested samples under different rotational speeds and side loads. At a constant load of 1000 N, samples tested at rotational speeds of 65 rpm and 115 rpm in Figure 8a,b, respectively, exhibited noticeably different wear characteristics. The sample tested at 115 rpm showed a larger worn area and more pronounced material detachment, corresponding to the increased wear depth, as previously explained in Figure 7c. Similarly,Figure 8c,d shows that the sample tested at 500 N displayed limited material removal, while the sample tested at 1000 N showed severe surface degradation, characterized by extensive matrix cracking, delamination, and the coating being worn out. This increased damage at a higher load is attributed to elevated surface temperatures, which accelerate matrix softening and degradation.

### 3.3. Specific Wear Rate

The specific wear rate K (10^−8^ MPa^−1^) of the composite casing coating was calculated using the following equation [38]:

K = V/(P × L)(1)

L = π × N × t × D(2)

where

V = WV × Sample width

where V is the wear volume (mm^3^), D is the tool joint diameter (mm), N is the rotational speed (rpm), P is the side load (N), t is the testing time (min), L is the total sliding distance (mm), and WV (mm^3^/mm) is the wear volume per unit width.

Table 3 presents the specific wear rate K for all tested side loads and rotational speeds. Each test was performed two to three times to ensure the repeatability and accuracy of the results. The K values were estimated at the end of the 5 h test for most samples. However, for samples tested under severe wear conditions (e.g., 1000 N/115 rpm, 1000 N/154 rpm, and 700 N/154 rpm), the specific wear rate (K) was calculated based on a sliding distance of 3 km, which corresponded to the functional end of the coating layer. Although the coating thickness was initially measured between 2.5 and 3.0 mm, the recorded wear depths using a dial gauge were often lower than this value. This discrepancy is attributed to three factors: (1) the curved geometry of the internal pipe coating, which caused the dial gauge to capture axial displacement rather than the true radial wear depth; (2) variation in coating thickness across the curved samples, which led to some regions wearing through earlier than others; and (3) the instrumental uncertainty of the digital indicator itself, which may introduce up to 10% error in depth measurement. Additionally, visual cues such as burn marks, smoke emission, and frictional sound indicated complete coating removal during testing, which was confirmed by post-test images in Figure 8b,d in the middle of the worn zone. To avoid overestimating the coating life or incorporating wear data from the substrate GFRE casing, wear rate calculations were limited to the first 3 km for these conditions.

**Figure 9:**
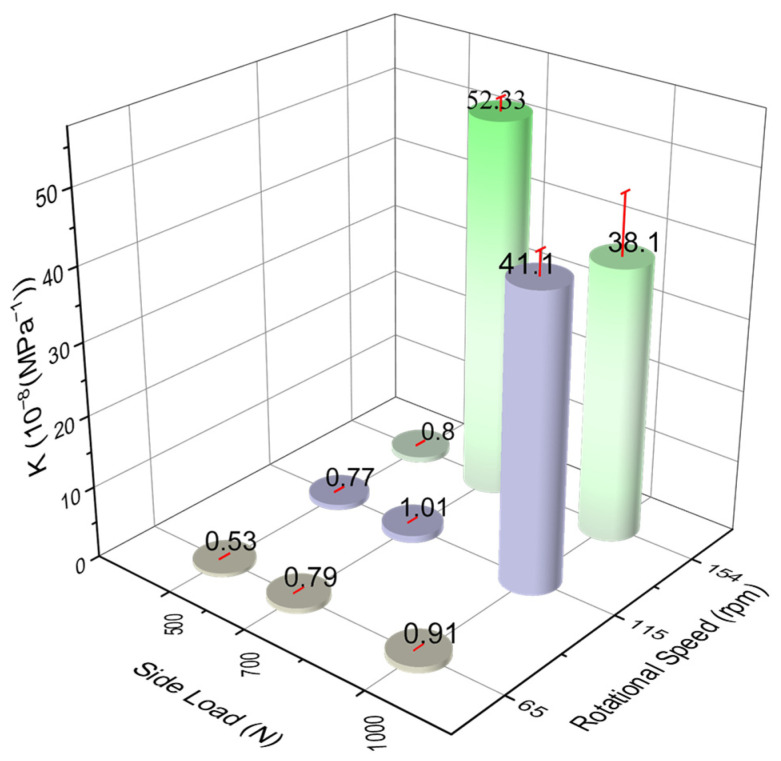
Average specific wear rate at different side loads and rotational speeds.

Figure 9 illustrates the average value of K under various side loads and DP-TJ rotational speeds. In general, the average K for most test conditions ranged between 0.43 and 1.0 × 10^−8^ MPa^−1^, with a noticeable increase with elevated side loads and rotational speeds. This trend is primarily attributed to the increase in frictional heat, which raises the bulk temperature at the composite surface, as depicted in Figure 7.

**Figure 10:**
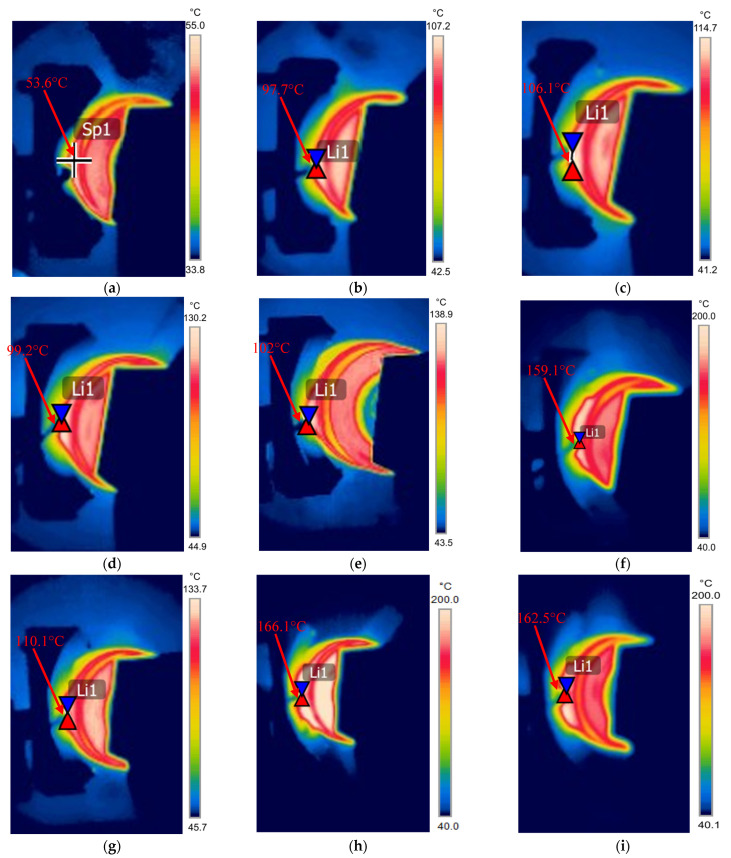
Thermal images at contact surface with indication of the highest temperature reached during the test: (**a**–**c**) at 65 rpm and 500, 700, and 1000 N; (**d**–**f**) at 115 rpm and 500, 700, and 1000 N; and (**g**–**i**) at 154 rpm and 500, 700, and 1000 N, respectively.

The thermal images from an infrared camera shown in Figure 10 also confirm the resulting temperature rise in the wear test zone. These images were used to validate the temperature readings obtained from the thermocouple and provide a clearer view of the temperature distribution around the contact zone, including an approximation of the contact temperature. The thermal contours clearly show that bulk temperature at the contact zone increased with both the rotational speed and side load in nearly all tested samples. The average specific wear rate for these conditions remained within the expected range for most composite linings and is consistent with the characteristic K factor values for epoxy-based composites [7,39].

Under high-wear conditions, the average specific wear rate increased dramatically to 41.1 × 10^−8^ MPa^−1^ at 1000 N and 115 rpm and 52.33 × 10^−8^ MPa^−1^ at 700 N and 154 rpm. In these cases, the coating was worn out in 50 to 66 min, as reported in Table 3. Based on the thermal images in Figure 10f,h,i, the contact temperature in these samples exceeded 160 °C, surpassing the glass transition temperature of the epoxy matrix. This explains the severe rise in the specific wear rate of these samples.

Interestingly, the sample tested at 700 N and 154 rpm showed a higher wear rate than the ones tested at 1000 N at both 115 and 154 rpm, which is unexpected. This could be due to the behavior of the wear debris generated from the SGFs, epoxy matrix, and nanofillers, which may act as a solid lubricant under dry sliding conditions [40]. At high side loads, the debris is more likely to be compacted at the contact surface due to elevated pressure, forming a third-body layer that separates the composite coating from the DP-TJ [31]. This layer reduces direct contact, limits adhesive wear, and helps stabilize friction. A detailed discussion on this is included in the following section.

**Figure 11:**
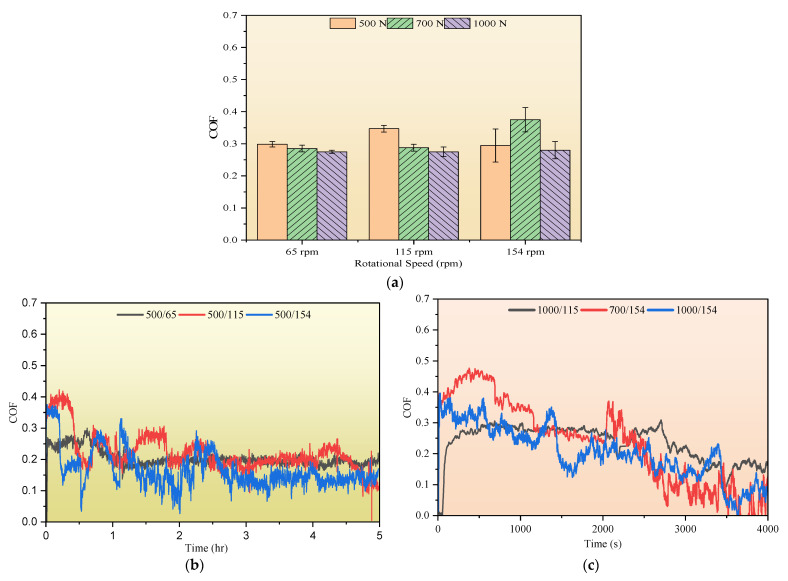
(**a**) Average coefficient of friction (COF) of the coating under different side loads and rotational speeds: (**b**) COF profiles during a 5 h test at a 500 N side load and rotational speeds of 65, 115, and 154 rpm and (**c**) COF profiles during a 4000 s test at 1000 N (115 and 154 rpm) and at 700 N (154 rpm).

Figure 11a presents the average coefficient of friction (COF) for the samples over the first 33 min of the wear test. These values are approximated using the force components recorded from the dynamometer. In general, the COF values ranged from 0.27 to 0.38. A slight increase in the COF was observed with increasing rotational speed, particularly under side loads of 700 N and 1000 N. The increase in the COF at 700 N and 154 rpm is likely attributed to the higher generation of matrix and fiber debris at elevated speeds. This debris, which contains SGFs and nanoparticles, accumulates at the sliding interface and acts as an abrasive third body, thereby contributing to increased friction. Figure 11b presents the COF profiles over the 5 h test duration at a 500 N side load for rotational speeds of 65, 115, and 154 rpm. At 65 rpm, the COF remains relatively stable, suggesting the formation of a stable tribofilm. This stability is likely due to a lower rotational speed, which allows wear debris to gradually compact and adhere to the surface, forming a protective third-body layer that reduces direct asperity contact. In contrast, at higher speeds (115 and 154 rpm), the increased rotational speed results in more fluctuation in the COF profile. Furthermore, Figure 11c presents the COF profiles at side loads of 1000 N (115 and 154 rpm) and 700 N (154 rpm), recorded over the first 4000 s of testing, after which the coating was fully worn. All conditions show fluctuating COF behavior but with distinct patterns. At 1000 N and 115 rpm, the COF increases gradually and remains relatively steady with mild oscillations. At 1000 N and 154 rpm, sharper fluctuations appear, particularly during the initial sliding, suggesting unstable frictional interactions. The 700 N and 154 rpm condition displays the highest COF variability, marked by frequent sharp increases and drops throughout the test duration. These variations in the COF profiles reflect differences in surface evolution and wear mechanisms under each combination of side load and rotational speed, which are further investigated through SEM and EDS analyses in the following section.

### 3.4. Wear Mechanisms

**Figure 12:**
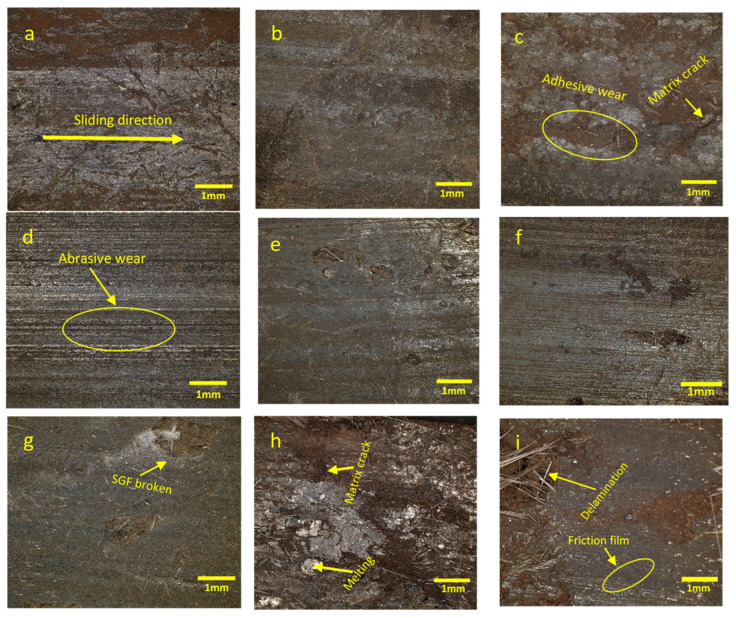
Microscopic images of worn surfaces at 65 rpm (**a**–**c**), 115 rpm (**d**–**f**), and 154 rpm (**g**–**i**) under side loads of 500 N, 700 N, and 1000 N, respectively.

The morphology of the worn surfaces obtained using a digital microscope revealed distinct wear mechanisms influenced by the rotational speed and side load. At a low rotational speed, adhesive wear is the predominant mechanism under all side loads, as evident in Figure 12a–c, where material transfer and localized bonding between the composite and DP-TJ are observed. Some matrix cracking is present in all three micrographs. As the rotational speed increases from 65 to 115 rpm and further to 154 rpm, a combination of abrasive and adhesive wear becomes apparent (Figure 12d–g), indicating increased material removal due to increased frictional heat and softening of the matrix, as discussed earlier. Severe wear degradation of the surface is observed at maximum rotational speed and under high side loads of 700 N and 1000 N. Figure 12h,I show excessive adhesive wear and delamination. Under these extreme conditions, matrix degradation is evident, leading to glass fiber peeling off, exposing fresh fiber surfaces and accelerating material loss.

Nanoparticles in epoxy composites have been shown to modify wear mechanisms by altering the interaction between the composite and the counterface, leading to improved tribological performance [41,42,43]. The wear behavior of SGF-reinforced epoxy composites is influenced by various mechanisms depending on material properties and wear conditions. One primary wear mechanism is matrix wear, which occurs due to plastic deformation under repeated stress, causing surface roughening and microcrack formation that weakens load transfer between the fibers and the matrix [44]. As these microcracks propagate over time, they result in delamination and further material loss, accelerating wear degradation. Another critical aspect is fiber wear, which includes multiple processes such as fiber sliding wear [45], where continuous friction between fibers and the counterface part leads to progressive surface degradation which increases the COF, resulting in higher heat generation. In addition, excessive mechanical loading can cause fiber cracking and crushing, resulting in fragmentation, which contributes to more exposure of fresh fiber surfaces, which exacerbates wear progression, as noticed in Figure 12h,i. A severe form of fiber degradation is fiber peeling off, which occurs due to weak fiber–matrix adhesion, leading to fiber detachment under wear stress and further material loss [46].

The combination of SiC, Al_2_O_3_, and CaCO_3_ nanoparticles in the epoxy composite coating is expected to contribute synergistically to the formation and stability of the tribofilm. SiC enhances the hardness and abrasion resistance of the coating by providing a rigid load-bearing phase that limits matrix removal during sliding [47]. Al_2_O_3_ supports thermal stability, helping the tribofilm maintain its integrity under friction-induced heat [48]. CaCO_3_, although softer, contributes during the early stages of sliding by filling microvoids, reducing surface roughness, and aiding in the initial formation of a smoother, more compact tribolayer [49]. Collectively, these nanofillers assist in the development of a durable and stable tribofilm, which plays a critical role in controlling friction and wear under varying load and speed conditions.

**Figure 13:**
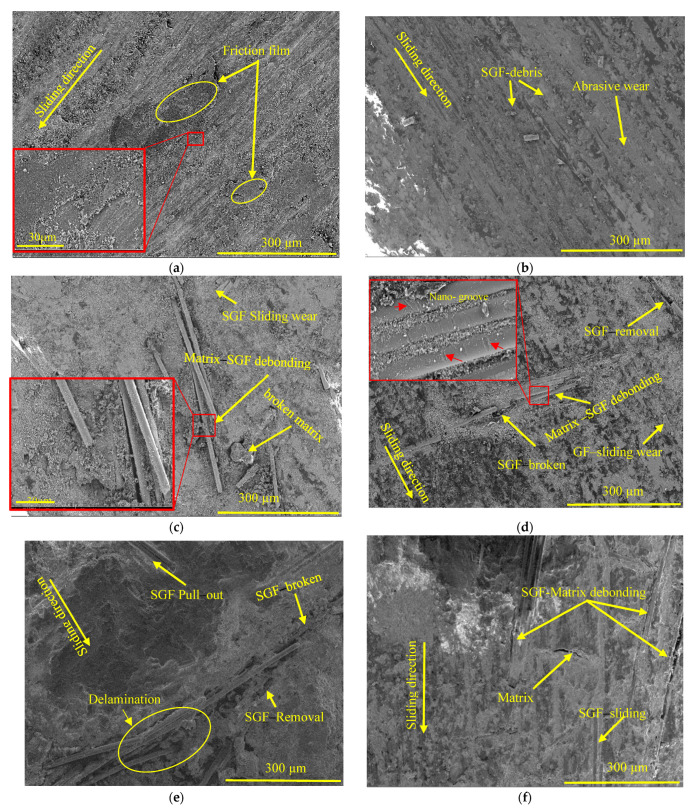
SEM images of the worn sample surfaces at (**a**) 500 N-65 rpm, (**b**) 500 N-115 rpm, (**c**) 700 N-65 rpm, (**d**) 500 N-154 rpm, (**e**) 1000 N-65 rpm, and (**f**) 700 N-115 rpm.

The SEM analysis of the worn surfaces in Figure 13 substantiates the discussed wear mechanisms influenced by variations in the rotational speed and side load. At low rotational speed and moderate side loads (e.g., 500 N, 65 rpm), the worn surfaces exhibit a relatively smooth morphology with the formation of a thin, uniform transfer film, as shown in Figure 13a. During sliding, fine debris generated from the epoxy matrix and dispersed nanoparticles accumulate at the contact interface. These particles gradually compact and fill surface irregularities, including the voids originally observed on the as-received epoxy composite surface, Figure 1b,c. This debris packing contributes to the formation of a continuous protective layer, effectively reducing surface roughness and minimizing direct contact between the composite and the counterface. At this stage, adhesive wear dominates, with no significant exposure or damage to the underlying glass fibers.

**Figure 14:**
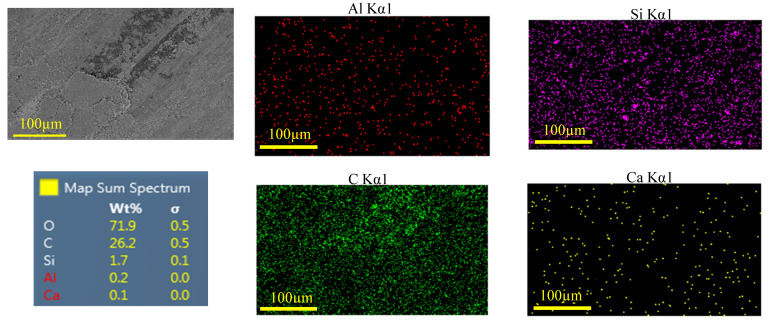
EDS analysis of the worn surfaces sample at 500 N-65 rpm.

EDS analysis of the worn surface of the sample in Figure 14 (65 rpm, 500 N) further supports this interpretation. The surface is predominantly carbon-rich, indicating matrix-derived debris, but also shows detectable levels of Si, Ca, and Al—suggesting that the incorporated nanoparticles (SiC, CaCO_3_, and Al_2_O_3_) were involved in the developing tribofilm. Their presence likely contributed to early-stage tribolayer formation by assisting in debris compaction, surface smoothing, and void filling. In particular, the detection of Ca supports the role of CaCO_3_ in reducing interfacial friction during the initial sliding phase. These observations are consistent with the smooth worn surface and stable COF behavior under these conditions, confirming that the nanoparticle system actively contributes to tribofilm formation and stability at lower loads.

As the rotational speed increases from 65 rpm to 115 rpm, under 500 N, Figure 13a,b, and 700 N, Figure 13c,f, the surface roughness increases, indicating a shift towards abrasive wear. The SEM images show an increased presence of large SGF debris on the surface which actively contributes to the abrasive wear process.

This transition from adhesive to abrasive wear is primarily due to higher frictional forces and localized heat generation, which softens the epoxy matrix and weakens its load-bearing capability. As a result, the glass fibers become increasingly exposed, sliding, thinning, and fragmented at the end due to higher shear and impact forces. The propagation of microcracks along the fiber length leads to brittle fracture and the formation of large-sized fragmented SGF debris, which further contributes to three-body abrasive wear [46].

At the highest rotational speed of 154 rpm under 500 N, the SEM analysis in Figure 13d reveals severe interfacial debonding, SGF breakage, and prominent SGF sliding wear. This behavior is attributed to the generated high temperatures (Figure 10), which cause the epoxy matrix to soften, reducing its ability to firmly retain the SGFs. As a result, the softened matrix wears off more rapidly, leading to increased exposure of the SGFs to direct contact with the DP-TJ, contributing to unstable tribofilm formation and fluctuations in the COF, as noted in Figure 11b. At high rotational speeds, the exposed fibers experience higher frictional forces, causing fragmentation of SGFs due to nano-grooves initiated by the effect of wear nanoparticle debris as third-body abrasive particles, as illustrated in Figure 13d. The continuous accumulation and circulation of these hard debris particles further enhance three-body abrasive wear, accelerating fiber breakage and promoting additional SGF fragments. This wear mechanism significantly reduces the composite’s structural integrity and increases the wear. Similarly, as shown in Figure 13a,c, increasing the applied load from 500 N to 700 N at 65 rpm results in noticeable interfacial debonding and large matrix fractures. This is primarily due to the excessive stress experienced by the epoxy matrix, leading to plastic deformation, microcrack formation, and eventual failure. As the matrix weakens, it loses its ability to effectively transfer stress, leading to stress concentration at the fiber–matrix interface and subsequent fiber debonding, pull-out, and peeling off. These mechanisms progressively reduce the composite’s wear resistance [50].

On the other hand, at a higher side load of 1000 N and lower rotational speeds, Figure 13e, the contact pressure significantly increases, further promoting crack propagation and ultimately leading to delamination. This catastrophic failure severely impairs the composite’s structural performance. Additionally, high temperatures exacerbate this degradation, as the softened epoxy matrix loses its mechanical strength and adhesion to fibers. The weakened matrix is more susceptible to wear, further accelerating interfacial debonding and fiber pull-out.

**Figure 15:**
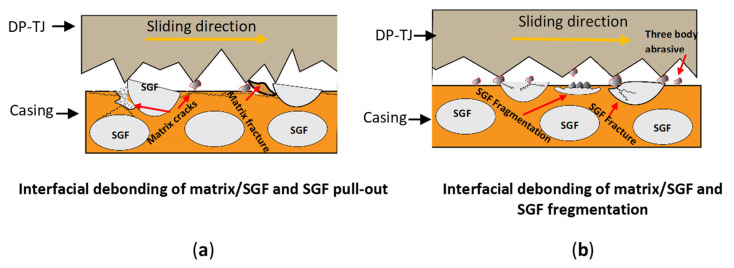
Schematic representation of the wear mechanisms of SGFs under the effects of (**a**) increasing side load and (**b**) increasing rotational speed.

Figure 15a schematically illustrates the wear mechanism of SGF epoxy composites under increasing side loads, while schematic Figure 15b demonstrates the effect of increasing the rotational speed. At higher side loads, matrix cracking occurs, leading to matrix fracture and interfacial debonding, ultimately resulting in the pull-out of the SGFs. Conversely, higher rotational speeds generate higher amounts of wear debris—originating from SGFs and nanoparticles—which acts as a third-body abrasive experiencing accumulation and circulation between the coated surface and DP-TJ surface. This debris contributes to the fragmentation of glass fibers, embedding within the matrix or counterface, further intensifying the abrasive wear mechanism.

**Figure 16:**
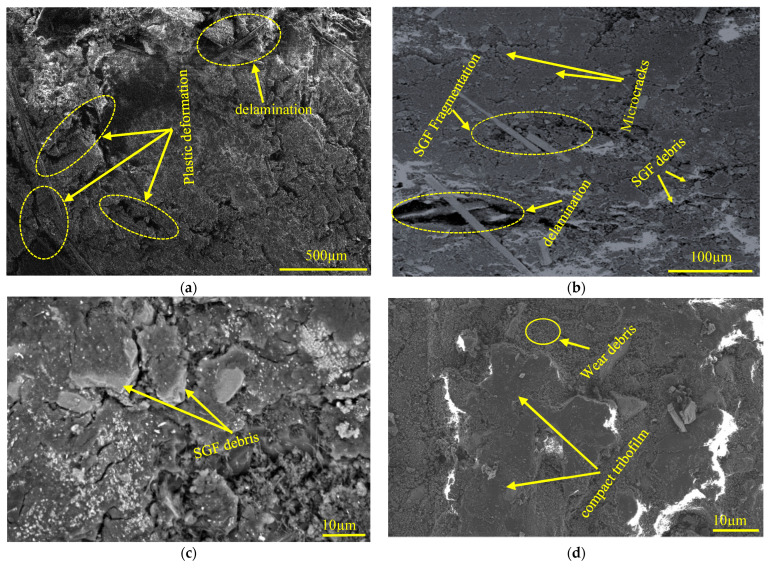
SEM images of the worn coating surfaces tested at (**a**) 700 N-154 rpm, (**b**) 700 N-154 rpm, (**c**) 700 N-154 rpm, and (**d**) 1000 N-115 rpm.

Under high-rotational-speed and high-side-load conditions (1000 N-115 rpm, 700 N-154 rpm, and 1000 N-154 rpm), the combined effects of elevated rotational speeds and side loads result in a severe wear mechanism, as observed in the SEM images in Figure 16. The sample with highest specific wear rate (700 N and 154 rpm) demonstrated significant plastic deformation and delamination of the epoxy matrix, which can be attributed to the intense frictional heat generated at the sliding interface. This thermal buildup raises the surface temperature, and once it exceeds the glass transition temperature (T_g_) of the epoxy resin (~120–150 °C), the matrix transforms from a rigid glassy state to a softened, rubbery phase. In this condition, the matrix loses its stiffness and load-bearing capacity, making it highly susceptible to deformation and wear under shear and compressive forces.

Simultaneously, the high contact pressure induced by the load promotes crack initiation and propagation within the weakened matrix, as seen in the SEM image in Figure 16b. These cracks concentrate energy at their tips, leading to matrix fracture and the subsequent exposure of the reinforcing SGFs. Once exposed, the fibers endure elevated temperatures and mechanical stresses, leading to brittle fracture, fiber pull-out, and fiber fragmentation.

**Figure 17:**
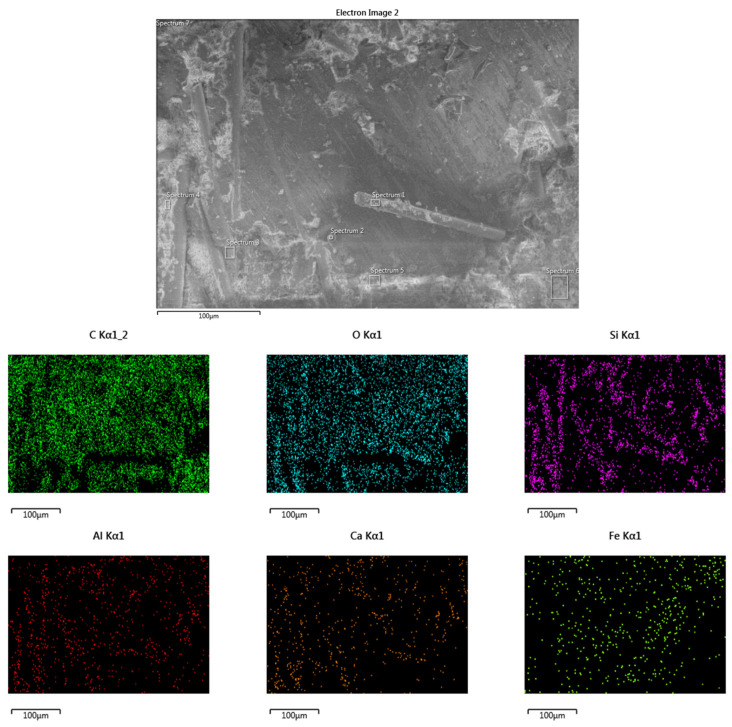
EDS mapping and elemental distribution at the top of a worn sample (700 N-154 rpm).

**Figure 18:**
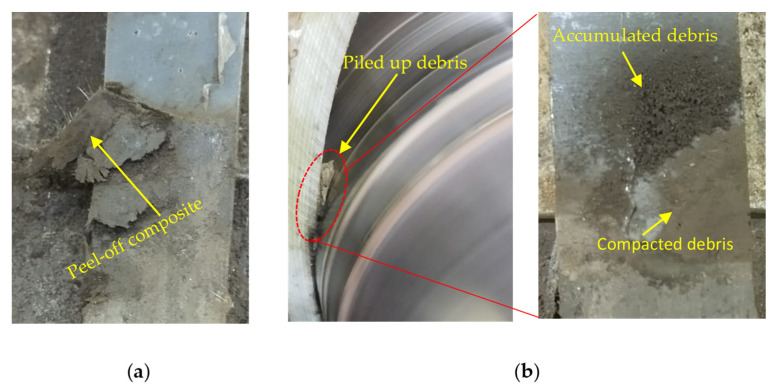
Photographic images showing (**a**) detachment of the composite coating and (**b**) debris accumulation.

The EDS mapping analysis in Figure 17 and Table 4 for the 700 N and 154 rpm sample revealed a notable presence of Fe on the worn surface—originating from the DP-TJ—as well as elevated levels of Si, Al, and Ca. These elements correspond to the presence of exposed SGFs and SiC, Al_2_O_3_, and CaCO_3_ nanoparticle phases on the worn surface, indicating severe wear and debris transfer. The synergistic effect of frictional heat and mechanical load at high rotational speeds and side loads led to layers of the composite material peeling off, as shown on the surface of the worn sample in Figure 18a at 1000 N-154 rpm, forming distinct debris accumulation which contributes to an increase in the specific wear rate.

**Figure 19:**
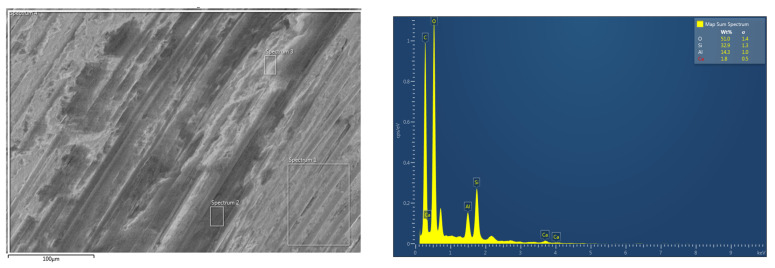
SEM image and EDS analysis spectrum of the top of a worn sample tested at 1000 N-115 rpm.

Additionally, the accumulation of wear debris was observed along the upper region of the wear track under high-speed, high-load conditions, Figure 18b. This debris becomes trapped within the contact interface, facilitating the formation of a compact tribofilm. However, under elevated pressure, the tribofilm becomes more compact and continuous. This supports the previously noted observation that at 700 N and 154 rpm, the specific wear rate increased to 52.33 × 10^−8^ MPa^−1^, which is higher than the values recorded at the higher load of 1000 N at 115 rpm and 154 rpm, where the specific wear rates were 41.1 × 10^−8^ MPa^−1^ and 38.06 × 10^−8^ MPa^−1^, respectively. The SEM micrographs in Figure 16c,d illustrate a clear difference in the surface conditions between the two loads. At 700 N, the worn surface appears rougher and is covered with loosely compacted, large-sized SGF fragments, indicating ineffective consolidation of the third-body layer. An increase in the shore D hardness reported earlier also confirms this observation. In contrast, the 1000 N sample exhibits a smoother worn surface with the presence of a compact, continuous tribofilm and finer debris. This suggests that the higher pressure at 1000 N further fragmented the SGFs and nanoparticles, enabling effective compaction and sintering of the wear debris into a stable protective layer. This interpretation is supported by the EDS mapping and spot analysis. At 1000 N, no Fe element was detected on the worn surface (Figure 19), indicating that the tribofilm shields the counterface surfaces from severe wear by preventing direct frictional contact and limits the excessive degradation of SGFs by filling the surface with compacted debris. In contrast, EDS analysis of the 700 N surface revealed significant Fe content (Table 4), confirming debris transfer from the steel counterface, which is consistent with an unstable and non-continuous tribolayer. While the worn surface at 700 N/154 rpm showed higher Ca content than at 1000 N/115 rpm (Table 4), this likely reflects CaCO_3_’s limited load-bearing capacity under high-pressure conditions. Due to its softer nature, CaCO_3_ contributes less to tribofilm stability at higher loads, where the formation of a compact and protective tribolayer is primarily governed by the harder SiC and thermally stable Al_2_O_3_ particles.

The COF behavior previously illustrated in Figure 11c aligns with these observations. The COF profile at 700 N displays strong fluctuations, indicative of unstable tribofilm formation and breakdown events. In contrast, the COF profile at 1000 N is more stable, suggesting the presence of a persistent tribolayer. The wear debris compacted on the surface of the counterpart is further supported by the presence of sheet-like debris stacks adhered to the DP-TJ surface in Figure 18b and aligns with previously reported mechanisms for tribofilm formation in polymer matrix composites under severe loading conditions [51,52,53].

## 4. Conclusions

A parametric study on custom-designed wear test apparatus simulating realistic drilling contact conditions was performed on a novel short-glass-fiber-reinforced epoxy composite coating with hybrid nanofillers (SiC, Al_2_O_3_, CaCO_3_) under contact with a hardened steel DP-TJ in dry conditions, and the following conclusions can be drawn:

1. The specific wear rate (K) and wear volume increased with both the rotational speed and side load. A significant rise in the average K value (52.33 × 10^−8^ MPa^−1^) was observed at speeds exceeding 115 rpm, particularly under intermediate loads (700 N), where the tribofilm was unstable and loosely compacted.
2. Under moderate conditions (500 N, 65 rpm), a stable tribofilm was formed, consisting of compacted debris including fragmented SGFs and nanofillers. This resulted in a low average K value of 0.53 × 10^−8^ MPa^−1^ and a stable COF, indicating effective protection by the composite coating.
3. At high loads of 1000 N, increased pressure enhanced debris compaction, leading to the formation of a continuous and dense tribofilm. This reduced direct contact and helped mitigate surface damage, even under severe sliding.
4. SiC and Al_2_O_3_ nanofillers played a critical role in enhancing tribofilm strength and stability under high-load abrasive conditions, while CaCO_3_ primarily contributed to filling surface voids and reducing interfacial friction under lower load conditions.
5. Under high-speed, high-load conditions, excessive frictional heat can soften the epoxy matrix into a viscous phase, triggering delamination and accelerating wear. The transition from abrasive to adhesive and delamination wear modes is strongly influenced by this thermal softening effect.

## Figures and Tables

**Table 1:** Dimensions of the coated casing and DP-TJ samples.

	Inner Diameter (mm)	Outer Diameter (mm)	Sample Width (mm)	Nonmetallic Composite Coating Thickness (mm)
Casing	200–310	245.6–355.6	16	2.5–3
DP-TJ	78–80	126	146	-

**Table 2:** Test matrix.

Test Condition	DP-TJ Rotational Speed (RPM)	Side Load (N)
1	65	500
2	65	700
3	65	1000
4	115	500
5	115	700
6	115	1000
7	154	500
8	154	700
9	154	1000

**Table 3:** Specific wear rate (K), maximum wear depth, and wear volume for casings.

Sample Code	Rotational Speed (rpm)	Side Load (N)	Time (min)	Wear Depth (mm)	Loss in Wear Volume (mm^3^)	Specific Casing Wear Rate K × 10^−8^ (MPa^−1^)
500/65	65	500	300	0.132	18.01	0.46
	65	500	300	0.155	22.87	0.59
	65	500	300	0.133	18.27	0.47
700/65	65	700	300	0.203	34.33	0.64
	65	700	300	0.257	48.67	0.90
	65	700	300	0.212	36.60	0.68
1000/65	65	1000	300	0.318	66.97	0.87
	65	1000	300	0.340	74.31	0.97
500/115	115	500	300	0.296	60.27	0.88
	115	500	300	0.244	45.11	0.66
700/115	115	700	300	0.423	102.82	1.01
	115	700	300	0.392	91.94	0.96
	115	700	300	0.421	102.08	1.07
1000/115	115	1000	66	2.550	1484.83	49.52
	115	1000	66	1.830	980.15	32.69
500/154	154	500	300	0.311	64.97	0.71
	154	500	300	0.260	49.76	0.55
	154	500	300	0.360	81.15	0.90
700/154	154	700	50	2.130	1146.04	53.83
	154	700	50	2.020	1082.27	50.83
1000/154	154	1000	50	2.380	1345.09	44.22
	154	1000	50	1.910	970.60	31.91

**Table 4:** EDX mapping and spot analysis results corresponding to SEM image in Figure 17.

Element	Overview Mapping (wt.%)	Spectrum 1 (wt.%)	Spectrum 3 (wt.%)	Spectrum 5 (wt.%)
O	45.3	49.5	48.4	24.4
Si	28.4	32.4	30.9	-
Al	12.2	9.1	10.0	-
Ca	7.8	5.8	10.7	-
Fe	6.3	3.2	-	31.9

## Data Availability

Data are contained within the article.
